# Editorial: Performance in Theatre and Everyday Life: Cognitive, Neuronal, and Applied Aspects of Acting

**DOI:** 10.3389/fpsyg.2021.732233

**Published:** 2021-08-11

**Authors:** Corinne Jola, Pil Hansen

**Affiliations:** ^1^Division of Psychology and Forensic Sciences, Abertay University, Dundee, United Kingdom; ^2^School of Creative and Performing Arts, University of Calgary, Calgary, AB, Canada

**Keywords:** acting, resilience, cognition, empathy, transfer effects, ecological validity, nondisciplinary research, social interaction

Performing an act involves complex cognitive and emotional processes that are not yet well understood. The aim of this Research Topic was thus to bring together empirical studies that address these within the realms of the many opportunities role-playing and acting provide. Whilst the prototype of acting is known as playing the role of somebody other than oneself for a non-performing audience, we all perform and act in everyday life. We may explicitly impersonate a character when, for example, reading bed-time stories, but we also, and more implicitly, perform a “social role.” Furthermore, something as simple as giving directions requires the ability to navigate a remembered scenario and anticipate future actions. If we acknowledge that these processes have performative functions, it is evident that acting and role-playing constitute an important part of human social behaviour. They are also found to develop early: pretend play is rehearsed as soon as a child has the capacity to understand that other people have different perceptions of events and contexts (i.e., theory of mind). Understanding the cognitive processes and underlying neuronal functions of theatrical performance can therefore be of great empirical and applied value to a variety of topics across disciplines.

The submitted works show that theatre offers itself to *interdisciplinary investigations* whilst facing *discipline-specific challenges*. The collection features two theoretical papers (Brown; McDonald et al.) and seven empirical studies (4 experimental and 3 survey-based) which are situated between theatre and the fields of atypical child development (Ioannou et al.), leisure (Pestana et al.) or education (Goldstein et al.; Hansen et al.; Schmidt et al.) and study topics such as perception and affect (Berceanu et al.; Olenina et al.). Considering the predominantly experimental stance of cognition and neuroscience research, one could argue that experimental designs are still underrepresented in studies of theatre. One reason is that studying cognitive and neuronal processes happens within the framework of *scientific reductionism*. As highlighted by Berceanu et al., it is difficult to dissect performance and plays into smaller parts without losing specific characteristics. Comparable issues are present in the field of dance (e.g., Christensen and Jola, 2015; Hansen, 2017), where a focus on quantifiable motion parameters can seemingly alleviate intangible facets. Similarly, elements such as language, gestures and poses seem to offer useful parameters for the study of acting, though examples remain limited. Within this topic, Berceanu et al. employed language as an indirect measure of how theatrical aggression is experienced, and Olenina et al. engaged participants in distilled theatrical poses to measure affective responses. These studies offer preliminary examples of how scientifically measurable causal relationships may be extracted from complex acting practices.

The study by Berceanu et al. showed that performing repetitive aggressive behaviour impacts participants' cognitive processes more strongly than passively watching performed aggression. Interestingly though, the actors reported more positive affect when performing aggressive acts than when watching them. This result may reflect a *coping mechanism* that involves emotional distancing. It is noteworthy that the survey-based study by Schmidt et al. also report a coping mechanism specific to acting students when comparing their empathic abilities with those of dance and psychology students. While all cohorts showed increased empathy compared to the general population, only acting students showed low levels of personal distress which can be a sign of resilience. Both studies indicate that experience in acting may foster coping mechanisms that separate action from emotion. Olenina's et al. experimental study, however, identified a link between action and affect: The authors examined effects on affect and high perception of expanding and contracting poses distilled from Michael Chekov's acting techniques. Based on their findings, the authors proposed that the two poses produce different affective states, while both forms of affect in turn alters the actors' egocentric spatial perception.

Theatre is an excellent means to study emotional responses and cognition in an ecological and ethically valid manner, and it is noticeable that three manuscripts in our topic have provided measurements in *ecologically-valid contexts* (Berceanu et al.; Hansen et al.; Ioannou et al.). However, these studies also have overlapping limitations: small sample sizes, lack of control group with comparable activity and/or lack of randomised assignment. These limitations reflect the ecological context as actors and performers work in small groups and are selected from a small pool. To advance while maintaining ecological validity, repetition of standardised experiments with multiple groups can increase sample sizes and the ability to arrange controls. As suggested by Hansen et al., mixed methods and analysis of mediating processes can also further query or qualify the statistical findings of small-sample studies.

The two submitted theoretical papers (Brown; McDonald et al.) focus on the *identification and classification of theatrical processes* within the plethora of empirical research. It is clear from these writings that work on building a common language across disciplines remains pertinent. Brown proposes a social “who” system, dealing with mentalising and pretence, in addition to the neuronally and functionally well-established “what,” “how,” and “where” systems. Social components are also among the focal points of the paper by McDonald et al., who reviewed skills involved in acting within neuroscientific topics such as memory, emotional control, empathy and theory of mind. The authors conclude that future studies should *consider specific performative techniques* as embodying characters physically - or generating them emotionally - will likely produce different observations. Our topic leans into this objective, as four submissions differentiate between performance techniques (Goldstein et al.; Ioannou et al.; Olenina et al.; Schmidt et al.).

Further, it is of interest to note that two of the survey-based studies focus on *the perceived impacts* of specific activities in theatre play aiming to better understand the mechanisms underlying change through theatre (Goldstein et al.; Pestana et al.). The latter's unique approach was to measure the impact of theatrical interventions as leisure activities on participants' sense of freedom and satisfaction. Group discussions and relaxation techniques were seen as most relevant for mindful self-processing. The former studied cause-and-effects of theatre training in school settings by investigating teachers' believes. Of importance were social interaction, perspective taking games, discussion of characterisations, performance in class and reflection on outcomes, such as empathy. These studies motivate research to determine whether believes match empirical effects.

Empirical insight into theatre and acting is needed to support more specific predictions and to target the application of *theatre exercises for health and learning*. Four studies in our topic address theatre with this aim (Goldstein et al.; Hansen et al.; Ioannou et al.; Schmidt et al.). For example, the experimental study by Ioannou et al. found that children and adults with intellectual functioning autism reported less anxiety and engaged in more group play with novel peers after a peer-mediated theatre intervention than a waitlisted group. Hansen et al. experimentally investigated the effect of systematic approaches to improvisation on executive functions and learning. The authors identified positive effects on inhibition, problem-solving, and cognitive flexibility. Qualitative analysis of participants' daily reports revealed learning challenges and cognitive demands encountered as well as transferable strategies devised to overcome them, such as re-directing attention.

With this topic we aimed to explore the state of empirical theatre research. We asked: (1) What do we know about how theatre works? and (2) What can theatre contribute to the understanding of the human brain and behaviour? The submitted manuscripts emphasise that theatre likely increases individuals' coping mechanisms and empathy and works through group discussions. Furthermore, specific acting exercises are associated with affect and enhance executive functions. Aspects of the self are theoretically pertinent, yet they remain empirically marginal. Methodological solutions are needed to advance beyond this state. In synthesis, the shared aim of the authors is to improve our understanding of the processes involved when controlling responses in social contexts and presenting the self in theatrical performances.

## Author Contributions

CJ wrote the first manuscript draft. PH completed the text and provided critical feedback. CJ and PH finalised the editorial together. All authors agreed to the final version.

## Conflict of Interest

The authors declare that the research was conducted in the absence of any commercial or financial relationships that could be construed as a potential conflict of interest.

## Publisher's Note

All claims expressed in this article are solely those of the authors and do not necessarily represent those of their affiliated organizations, or those of the publisher, the editors and the reviewers. Any product that may be evaluated in this article, or claim that may be made by its manufacturer, is not guaranteed or endorsed by the publisher.
